# Pyloric drainage interventions for gastroparesis: a comparison of laparoscopic pyloroplasty and gastric peroral endoscopic myotomy (G-POEM) outcomes

**DOI:** 10.1007/s00464-025-11731-3

**Published:** 2025-04-18

**Authors:** Sven E. Eriksson, Marie-Lise Chrysostome, Inanc S. Sarici, Johnathan Nguyen, Ping Zheng, Shahin Ayazi

**Affiliations:** 1 Foregut Division, Surgical Institute, Allegheny Health Network, 4815 Liberty Avenue, Suite 454, Pittsburgh, PA 15224 USA; 2 Chevalier Jackson Esophageal Research Center, Western Pennsylvania Hospital, Pittsburgh, PA USA; 3 Department of Surgery, Drexel University, Philadelphia, PA USA

**Keywords:** Gastroparesis, Pyloroplasty, G-POEM, Outcomes, Safety, Gastric-emptying scintigraphy

## Abstract

**Background:**

The endoscopic approach to the surgical management of gastroparesis is gaining popularity. However, data comparing endoscopic myotomy to traditional laparoscopic pyloroplasty are limited. This study aimed to compare outcomes between gastric-per-oral endoscopic myotomy (G-POEM) and pyloroplasty.

**Methods:**

Gastroparesis patients who underwent pyloroplasty or G-POEM from 2013 through 2023 at our institution were reviewed. Pre/postoperative gastroparesis cardinal symptom index (GCSI), resolution of the predominant gastroparesis symptom, and gastric emptying scintigraphy (GES) were assessed.

**Results:**

There were 314 patients who underwent surgical myotomy. Median (IQR) age was 51.9 (40–62) and 84.1% were female. Of these 81 underwent G-POEM and 233 underwent pyloroplasty. Age and sex were similar between surgical approaches (*p* > 0.05). Gastroparesis etiology was 61.8% idiopathic, 21.3% diabetic, and 16.9% postsurgical. Postsurgical etiology was more likely to undergo G-POEM (25.9% vs. 13.7%, *p* = 0.046). At a mean (SD) of 14.2(17) months resolution of the predominant gastroparesis symptom was achieved by 70.0% after G-POEM and 76.4% after pyloroplasty (*p* = 0.297). The GCSI improved after G-POEM [3.1(2–4) to 2.4 (2–3), *p* = 0.0498] and pyloroplasty [3.1(3–4) to 2.4(2–3), *p* < 0.0001]. There was no difference in postoperative GCSI (*p* = 0.805) or percent GCSI improvement (*p* = 0.976) between groups. In the 96 patients with pre- and postoperative GES, 4 h retention decreased for pyloroplasty from 29.0% (18–43) to 4.2% (1.0–18) (p < 0.0001) and for G-POEM from 23.0 (13–49) to 13 (5.0–29), (*p* = 0.045). Pyloroplasty showed a trend towards better emptying (*p* = 0.0719) with more patients achieving ≥ 50% improvement (70.3% vs. 50%, *p* = 0.086).

**Conclusions:**

Gastroparesis symptom improvement was similar after pyloroplasty and G-POEM; however, there was a trend towards better improvement in gastric emptying after pyloroplasty.

**Graphical abstract:**

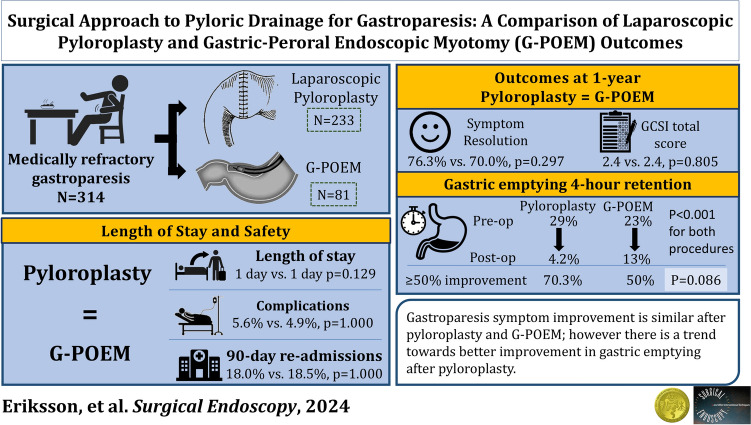

The management of gastroparesis is complex and nuanced. The non-specific upper gastrointestinal symptoms of nausea, vomiting, early satiety, bloating and abdominal pain, and the plurality of etiologies associated with gastroparesis complicate both diagnosis and assessment of effective treatments. Metoclopramide remains the only FDA approved medication for gastroparesis. It is typically prescribed in conjunction with anti-emetics and dietary modifications, but these therapies are not uniformly effective. There is no broadly agreed upon consensus definition of medically refractory gastroparesis; however, the American Gastroenterological Association (AGA) has defined it as persistent non-medication-induced symptoms in the context of objectively confirmed gastric-emptying delay without mechanical obstruction and despite the use of dietary adjustment and metoclopramide [1]. As gastroparesis symptoms incur a markedly detrimental effect on patient quality of life, many turn to alternative treatments such as surgical intervention for medically refractory gastroparesis [2]. However, there has been a paucity of robust data in the literature to guide the management of medically refractory gastroparesis. Guidelines do not make any specific recommendations as to when surgical intervention is appropriate over other modalities; only that surgery is better than no treatment [1, 3].

Originally used for the management of pyloric outflow obstruction, pyloric drainage surgery has emerged as an effective surgical intervention for medically refractory gastroparesis over the past few decades. The mechanism of pyloric drainage is incompletely understood. However, it is thought to ameliorate ‘pylorospasm’ and address discordant antral contractility without pyloric relaxation seen in some patients with gastroparesis. Meta-analyses have demonstrated that pyloric drainage surgery improves symptoms and enhances gastric emptying in more than 75% of patients with medically refractory gastroparesis [4]. However, the majority of studies are limited in sample size and follow-up. There is a need for better powered studies with longer-term outcomes. The two most studied approaches to pyloric drainage surgery are laparoscopic pyloroplasty and gastric peroral endoscopic myotomy (G-POEM). Pyloroplasty involves making a full-thickness longitudinal incision through the pylorus and then suturing it in a transverse fashion to widen the opening. The G-POEM is a more recent minimally invasive endoscopic procedure that uses submucosal tunneling to perform a pyloromyotomy.

It has been suggested that the advanced endoscopic techniques used in G-POEM may confer similar outcomes to laparoscopic pyloroplasty with a safer profile [5]. Limited independent studies have assessed the safety and efficacy of pyloroplasty or G-POEM separately. However, there is a paucity of studies that directly compare the two procedures in terms of safety, clinical success, or improvement in gastric emptying. Therefore, we designed this study to determine and compare the safety and efficacy of laparoscopic pyloroplasty and G-POEM for the improvement of symptoms and gastric emptying in patients with medically refractory gastroparesis.

## Materials and methods

### Study population

This is a retrospective review of prospectively collected data from patients who underwent either laparoscopic pyloroplasty or G-POEM for the management of medically refractory gastroparesis at Allegheny Health Network hospitals (Pittsburgh, PA) between 2013 and 2023. Demographic, clinical, and objective testing data were collected and reviewed, including gastroparesis etiology type (idiopathic, diabetic, or postsurgical), gastric-emptying scintigraphy (GES) results, predominant gastroparesis symptom and gastroparesis cardinal symptom index (GCSI) questionnaires. Patients younger than 18 and those with a history of prior pyloric drainage surgery were excluded from analysis. This study was evaluated and approved by the Institutional Review Board of Allegheny Health Network (IRB 2020-076).

### Gastroparesis diagnosis and assessment

Prior to surgery, all patients underwent a detailed clinical foregut evaluation and clinically indicated objective testing, with a focus on documentation of delayed gastric emptying, identification of gastroparesis etiology (diabetic, postsurgical, or idiopathic), and exclusion of alternative diagnoses (e.g., rumination syndrome, cyclic vomiting syndrome, eating disorders, etc.). Patients were asked to report a most bothersome predominant gastroparesis symptom. The most frequently reported predominant symptoms were ‘Nausea/Vomiting,’ ‘Bloating,’ ‘Abdominal pain,’ and ‘Early satiety.’ Patients diagnosed with gastroparesis initially met with a specialized dietician to discuss dietary modifications and were medically optimized in terms of indicated prokinetics, anti-emetics, and comorbidities (e.g., diabetes, chronic pain management). Patients also completed the gastroparesis cardinal symptom index (GCSI) questionnaire [6]. The GCSI is a patient self-reported questionnaire assessing 9 symptoms of gastroparesis on a Likert scale ranging from 0 (none) to 5 (very severe symptoms). Ratings on the 3 symptom items related to nausea, retching and vomiting are averaged to calculate the nausea/vomiting sub-score. The early satiety sub-score is the mean of 4 symptom items related to stomach fullness, inability to finish a normal-sized meal, excessive fullness, and loss of appetite. The bloating sub-score is the mean of 2 symptom items related to bloating and distention. The GSCI total score was calculated by taking the mean of these 3 sub-scores as previously described [6]. Assessment of the presence of the patient’s predominant gastroparesis symptom and the GCSI questionnaire was completed preoperatively, and at follow-up after surgical intervention.

### Gastric emptying scintigraphy technique and interpretation

Patients ingested a standardized meal containing 1 mCi of technetium-99m sulfur colloid. A series of anterior and posterior images were taken over the abdomen for 60 s immediately following ingestion, and then at hourly intervals for 4 h. The region containing the stomach was identified, and radiometric counts from this region immediately after ingestion were compared to the attenuation corrected counts at the hourly intervals to determine percent meal retention. A percent retention at 4 h > 10% was considered delayed gastric emptying.

### Laparoscopic pyloroplasty technique

After placement of the ports, the pylorus was identified and mobilized to allow a tension-free closure. A 4-cm full-thickness pyloric myotomy was made extending from the antrum to the duodenum using a harmonic scalpel. A Heineke–Mikulitz pyloroplasty was then performed as previously described [7]. Endoscopy was then repeated to evaluate luminal patency and perform a leak test. An upper GI contrast study was performed on postoperative day one to assess for contrast extravasation or obstruction.

### Gastric peroral endoscopic myotomy (G-POEM) technique

The technique was identical in all patients, and all procedures were performed under general anesthesia. A submucosal cushion was created approximately 2–3 cm proximal to the pylorus along the lesser curve. A transverse mucosal incision was made with a triangle tip knife. The endoscope was advanced into the submucosal tunnel, and the dissection was carried down to the pylorus. The pyloric muscular fibers were then divided. After completion, the surgical site was examined for serosal injury, irrigated, and the mucosal incision was closed with several Resolution™ clips (Boston Scientific, Marlborough, MA). An upper GI contrast study was performed on postoperative day one to assess for contrast extravasation or obstruction.

### Outcomes and statistical analysis

Demographic and preoperative clinical and objective testing data were compared between patients who underwent primary G-POEM or pyloroplasty for medically refractory gastroparesis. Surgical outcomes in terms of lengths of stay, readmissions, postoperative GCSI scores, need for subsequent gastric stimulator, and need for subsequent gastrectomy were assessed. Preoperative and postoperative GCSI scores were compared for each procedure group. Postoperative outcomes were compared between patients who underwent G-POEM or pyloroplasty. A subset of patients who completed pre- and postoperative GES were assessed for improvement in percent retention at 4 h, at least 50% improvement in 4 h retention and normalization. These GES outcomes were compared between procedures. Values for continuous variables are expressed as either mean (SD) or median with interquartile range where appropriate. Values for categorical variables are presented as frequency and percentage. Comparison analysis for two groups was performed by means of nonparametric tests, including Mann–Whitney test for difference, Pearson’s chi-square test, and Fisher’s exact test when appropriate. Comparison analysis for pre/post-Op measures was performed by type 3 test of fixed effect using a generalized linear mixed model, in which subject effect was included in the analysis. A *p* value < 0.05 was considered to be statistically significant. All statistical analyses were performed using SAS software (version 9.4, SAS Institute, Cary, NC).

## Results

The final study population consisted of 314 patients who underwent primary surgical or endoscopic myotomy for the management of medically refractory gastroparesis. The median (IQR) age was 51.9 (40–62) with a BMI of 27.6 (24–33) and 84.1% of patients were female. The most common predominant symptom was nausea/vomiting at 43.9%. The most common etiology was idiopathic (61.8%), followed by diabetic (21.3%) and postsurgical (16.8%). There were 233 patients (74.2%) who underwent laparoscopic pyloroplasty and 81 who underwent G-POEM. The demographic and preoperative clinical characteristics are compared between the laparoscopic and endoscopic procedures in Table 1. The groups were not significantly different across any measure, except patients who underwent G-POEM were more likely to report bloating as their predominant symptom than another symptom (30.9% vs. 18.0%, *p* = 0.018). Patients with a postsurgical etiology were more likely to undergo G-POEM (25.9% vs. 13.7%, *p* = 0.046).

**Table 1:** Comparison of demographic and preoperative clinical characteristics

	Pyloroplasty (*n* = 233)	G-POEM (*n* = 81)	*p* Value
Age, years, median (IQR)	51.4 (39–62)	53.9 (42–64)	0.276
Sex			
Male, %	13.7%	22.2%	0.080
Female %	86.3%	77.8%	
BMI, kg/m^2^, median (IQR)	27.5 (24–3.2)	28.1 (25–33)	0.134
Gastroparesis type			
Diabetic	22.3%	18.5%	0.0461
Idiopathic	63.9%	55.6%	
Postsurgical	13.7%	25.9%	
Predominant gastroparesis symptom			
Nausea/vomiting	55.4%	53.1%	0.056
Bloating	18.0%	30.9%	
Abdominal pain	17.6%	12.3%	
Early satiety	9.0%	3.7%	
Gastroparesis cardinal symptom index (GCSI)			
Total score, median (IQR)	3.1 (3–4)	3.1 (2–4)	0.624
Nausea/vomiting score, median (IQR)	2.3 (1–4)	3.0 (1–4)	0.947
Early satiety score, median (IQR)	3.8 (3–4)	3.3 (3–4)	0.197
Bloating score, median (IQR)	4.0 (3–4)	3.5 (2–4)	0.604

*G-POEM* gastric-per-oral endoscopic myotomy, *IQR* interquartile range, *BMI* basal metabolic index, *GI* gastrointestinal, *GCSI* gastroparesis cardinal symptom index

### Pyloroplasty outcomes

After pyloroplasty patients stayed in the hospital for a median (IQR) of 1 (1–2) day, with 72.1% of patients discharged on postoperative day 1. There were 30 (12.8%) readmissions within 30 days and 42 (18.0%) readmissions within 90 days of surgery. A Clavien-Dindo Grade III or higher complication developed in 5.6% of patients after pyloroplasty. These included leaks detected on postoperative day (POD) 1 requiring reoperation and omental patch repair (2 events); intrabdominal abscess requiring readmission for drainage, despite no evidence of a leak on POD 1 upper GI contrast study (1 event); intractable nausea/vomiting with dehydration requiring EGD dilation (4 events); deep vein thrombosis/pulmonary embolism (2 events); postoperative hypoxemia requiring continued ventilator support (2 events) in one patient with severe COPD and one patient with a BMI > 45 asthma and sleep apnea, one patient developed an intraoperative air embolus complicated by patent foramen ovale and stroke; and one patient developed a pyloric hematoma requiring reoperation (1 event). In addition, 2.6% of patients developed surgical site infections.

At a mean follow-up of 15.5 (19) months after pyloroplasty, there was a significant decrease in the GCSI total score [3.1 (3–4) to 2.4 (2–3), *p* < 0.0001], vomiting sub-score [2.3 (1–4) to 1.3 (1–3), *p* < 0.0001], early satiety sub-score [3.8 (3–4) to 2.8 (2–4), *p* < 0.0001], and bloating sub-score [4.0 (3–5) to 3.0 (2–4), *p* = 0.0004].

Additional surgical intervention for refractory gastroparesis was required for 13.3% of patients after pyloroplasty. There were 25 (10.7%) patients who underwent subsequent gastric stimulator implantation at 22.3 (11.8) months after the index operation. There were 6 (2.6%) patients who underwent gastrectomy at 28.5 (21.6) months after index operation.

### G-POEM outcomes

After G-POEM patients stayed in the hospital for a median of 1 (1–1) day, with 85.2% of patients discharged on postoperative day 1. There were 9 (11.1%) readmissions within 30 days and 15 (18.5%) readmissions within 90 days of surgery. Clavien–Dindo Grade III or higher complication developed in 4.9% of patients after G-POEM. One patient, who was on chronic immunosuppressants for lupus, presented on POD 14 with an abscess requiring drainage despite no evidence of leak on POD 1 contrast study. There were two patients who presented with intractable nausea/vomiting with dehydration requiring EGD dilation. One patient developed bleeding at the mucosotomy site on POD 1 requiring endoscopic intervention.

At a mean follow-up of 10.5 (12) months after G-POEM, there was a significant decrease in the GCSI total score [3.0 (2–4) to 2.4 (2–3), *p* = 0.0498]. The vomiting sub-score also significantly improved [3.0 (1–4) to 1.7 (1–3), *p* = 0.034]. Early satiety [3.3 (3–4) to 2.8 (2–4), *p* = 0.194] and bloating [3.5 (3–4) to 3.0 (2–4), *p* = 0.179] did not significantly decrease.

Additional surgical intervention for refractory gastroparesis was required for 8.6% of patients after G-POEM. There were 6 (7.4%) patients who underwent subsequent gastric stimulator implantation at 7.0 (4.8) months after the index operation. There were 2 (2.5%) patients who underwent gastrectomy at 15.1 (14.9) months after index operation.

### Comparison of pyloroplasty and G-POEM outcomes

**Fig. 1:**
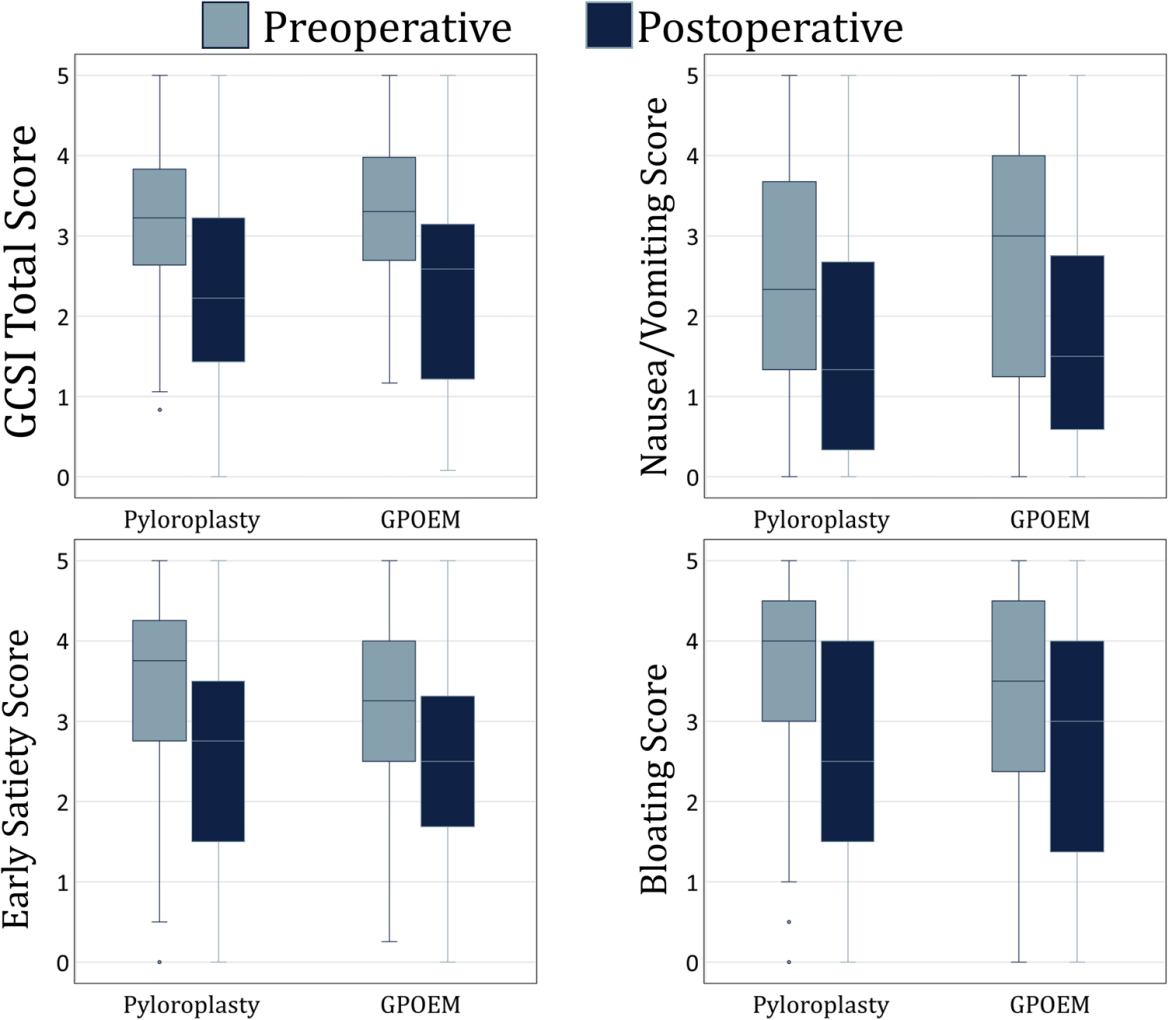
Box whisker plots of the median (IQR) GCSI total score and sub-scores before (blue) and after (orange) either laparoscopic pyloroplasty or G-POEM. There were no differences between pyloroplasty and G-POEM across all scores (*p* > 0.05) (Color figure online)

Follow-up times were similar between pyloroplasty and G-POEM (*p* = 0.107). Surgical outcomes are compared between pyloroplasty and G-POEM in Table 2**.** There was no difference in average length of stay (*p* = 0.129); however, patients who underwent G-POEM were more likely to be discharged on postoperative day 1 (85.2% vs. 72.1%, *p* = 0.024) Readmission rates and complication rates were also similar. There was no difference in resolution of predominant gastroparesis symptoms even when stratified by gastroparesis etiology (Table 3). There was no difference in GCSI total scores or any symptom sub-scores (Fig. 1). The median percentage GSCI total score improvement was 37% (5–66) in patients who underwent G-POEM and 36.5% (8–59) in patients who underwent pyloroplasty (*p* = 0.976). There was no difference in need for gastric stimulator (*p* = 0.517) or gastrectomy (*p* = 1.000). There were no mortalities in either group.

**Table 2:** Comparison of G-POEM and pyloroplasty outcomes

	Pyloroplasty (*n* = 233)	G-POEM (*n* = 81)	*p* Value
Length of stay, median IQR, days	1 (1–2)	1 (1–1)	0.129
Resolution of predominant symptom, %	76.4%	70.0%	0.297
Resolution of predominant nausea/vomiting, %	73.4%	62.5%	0.232
Resolution of predominant early satiety, %	90.5%	66.7%	0.343
Resolution of predominant bloating, %	76.2%	76.0%	1.000
Resolution of predominant abdominal pain, %	95.1%	80.0%	0.168
GCSI total Score, median (IQR)	2.4 (2–3)	2.4 (2–3)	0.805
Nausea/vomiting score, median (IQR)	1.3 (0–3)	1.7 (1–3)	0.785
Early satiety score, median (IQR)	2.8 (2–4)	2.8 (2–4)	0.945
Bloating score, median (IQR)	3.0 (2–4)	3.0 (2–4)	0.875
Complication ≥ Clavian-Dindo Grade III, %	5.6%	4.9%	1.000
30 day readmission, %	12.8%	11.1%	1.000
90 day readmission, %	18.0%	18.5%	1.000
Need for gastric stimulator, %	10.7%	7.4%	0.517
Need for gastrectomy, %	2.6%	2.5%	1.000

*G-POEM* gastric-per-oral endoscopic myotomy, *IQR* interquartile range, *GCSI* gastroparesis cardinal symptom index

**Table 3:** Comparison of resolution of predominant symptom between G-POEM and pyloroplasty by gastroparesis etiology

	Pyloroplasty (*n* = 233)	G-POEM (*n* = 81)	*p* Value
Diabetic, *n*	52	14	
Resolution of predominant symptom, *n* (%)	45 (86.5)	9 (64.3)	0.111
Resolution of predominant nausea/vomiting, *n* (%)	28 (80.0)	7 (70.0)	0.668
Resolution of predominant early satiety, *n* (%)	4 (100.0)	1 (100.0)	–
Resolution of predominant bloating, *n* (%)	5 (100.0)	1 (50.0)	0.286
Resolution of predominant abdominal pain, *n* (%)	8 (100.0)	0 (0.0)	0.111
Idiopathic, *n*	148	44	
Resolution of predominant symptom, *n* (%)	112 (75.7)	31 (70.5)	0.555
Resolution of predominant nausea/vomiting, *n* (%)	55 (67.9)	15 (65.2)	0.806
Resolution of predominant early satiety, *n* (%)	13 (92.9)	1 (50.0)	0.242
Resolution of predominant bloating, *n* (%)	19 (73.1)	10 (76.9)	1.000
Resolution of predominant abdominal pain, *n* (%)	25 (92.6)	5 (83.3)	0.464
Postsurgical, *n*	32	20	
Resolution of predominant symptom, *n* (%)	27 (84.4)	14 (70.0)	0.299
Resolution of predominant nausea/vomiting, *n* (%)	11 (91.7)	3 (42.9)	0.038
Resolution of predominant early satiety, *n* (%)	2 (66.7)	–	–
Resolution of predominant bloating, *n* (%)	8 (72.7)	8 (80.0)	1.000
Resolution of predominant abdominal pain, *n* (%)	6 (100.0)	3 (100.0)	–

*G-POEM* gastric-per-oral endoscopic myotomy

### Gastric emptying before and after pyloric drainage

**Fig. 2:**
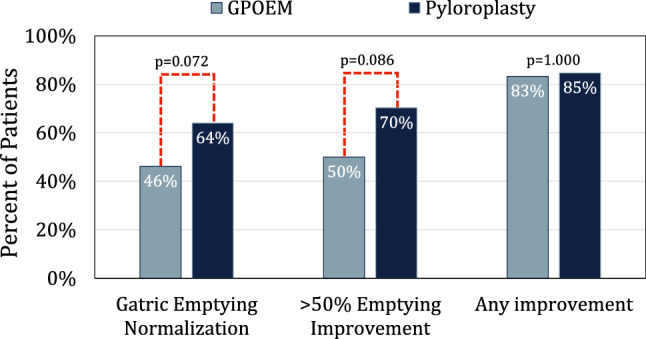
Percent of patients who achieved gastric-emptying scintigraphy (GES) normalization (< 10% retention at 4 h), who achieved > 50% improvement in % retention at 4 h, and who achieved any improvement in % retention at 4 h after pyloroplasty or G-POEM. There was a trend towards worse normalization (*p* = 0.072) and improvement (*p* = 0.086) after G-POEM. There was no difference in ‘any improvement’

Gastric emptying findings are compared between pyloroplasty and G-POEM groups in Table 4. Prior to surgery, the median percent retention of gastric contents 4 h after ingestion was 28.5% (17–46) with 22.1% of patients with severely delayed emptying (> 50% 4 h retention). There was no difference in 4 h retention (*p* = 0.307) or severely delayed gastric emptying (*p* = 1.000) between patients who underwent pyloroplasty or G-POEM. There were 96 patients (71 pyloroplasty; 25 G-POEM) who completed pre and postoperative GES. After pyloroplasty, 4 h retention decreased from 29.0 (18–43) to 4.2 (1.0–18), (*p* < 0.0001), with 84.7% GES improvement 64.0% GES normalization and 94.2% of patients free from severely delayed gastric emptying. After G-POEM, 4 h retention decreased from 23.0 (13–49) to 13 (5.0–29), (*p* = 0.045), with 83.3% GES improvement, 46.2% GES normalization, and 88.5% of patients free from severely delayed gastric emptying. There was no significant difference in gastric emptying between surgeries postoperatively. However, there was a trend towards worse 4 h retention, lower gastric-emptying normalization, and less GES improvement in patients who underwent G-POEM (Fig. 2).

**Table 4:** Comparison of gastric emptying between G-POEM and pyloroplasty

	Pyloroplasty	G-POEM	*p* Value
Preoperative			
4 h retention, %, median (IQR)	29.0 (18–43)	23.0 (13–49)	0.307
Severely delayed emptying, %	22.0%	22.5%	1.000
Postoperative			
4 h retention, %, median (IQR)	4.2 (1.0–18)	13 (5.0–29)	0.072
Severely delayed emptying, %	5.8%	11.5%	0.385
Percent change in 4 h retention, median (IQR)	84.1 (42–96)	60.3 (13–91)	0.164

*G-POEM* gastric-per-oral endoscopic myotomy, *IQR* interquartile range

## Discussion

Pyloroplasty has been part of the surgical practice since the late nineteenth century when it was introduced for the management of pyloric obstruction and peptic ulcer disease [8, 9]. The application of pyloric drainage surgery in gastroparesis emerged in the late twentieth century as improved gastric emptying and symptom relief were observed in patients with pyloric dysfunction. With the advent of minimally invasive surgery, laparoscopic pyloroplasty became as a preferred surgical option for medically refractory gastroparesis [10, 11]. More recently, G-POEM was developed as an endoscopic alternative, based on the success of the esophageal per-oral endoscopic myotomy (POEM) in treating achalasia [12]. G-POEM allows for targeted pyloric muscle disruption to enhance gastric emptying with reduced invasiveness, making it an appealing option for patients with gastroparesis. Both procedures are increasingly utilized for refractory gastroparesis, though direct comparative data remain limited, primarily consisting of small single-arm observational studies with short follow-up. To our knowledge, there are only two prior studies directly comparing pyloroplasty and G-POEM outcomes. In the present study, we evaluated 314 patients with medically refractory gastroparesis who underwent either laparoscopic pyloroplasty or G-POEM and found both procedures to be safe and effective. There were no substantial differences in length of hospital stay, readmissions, or complications, suggesting a comparable safety profile. In addition, there were no differences in symptomatic outcomes in terms of resolution of the predominant gastroparesis symptom, GCSI total scores, or the symptom sub-scores, suggesting comparable clinical efficacy. In a subgroup of patients who underwent postoperative GES testing, emptying substantially improved after both procedures, but there was a trend towards superior emptying after pyloroplasty.

The gastroparesis cardinal symptom index (GCSI) is the most frequently used validated questionnaire in the assessment of quantitative symptomatic improvement after surgical intervention for gastroparesis. We found a similar improvement in the GCSI scores between pyloroplasty and G-POEM. The literature also demonstrates similar improvement between the laparoscopic and endoscopic approaches. The G-POEM literature demonstrates that GCSI total scores improve from 2.1–3.8 preoperatively to 0.8–2.4 postoperatively with the mean improvement ranging from 1.1 to 2.5 points [13–24]. Similarly, the pyloroplasty literature demonstrates that GCSI total scores improve from 3.4–3.7 preoperatively to 1.3–2.3 postoperatively with a difference ranging from 1.6 to 2.1 points [10, 25–29]. These findings align with our results. However, existing studies are limited by small sample sizes—most with 30 or fewer patients—and short follow-up, with half of these studies reporting outcomes at ≤ 3 months. In contrast, the present study has a large sample size and demonstrated that from a symptomatic standpoint, G-POEM and pyloroplasty are equally effective options for medically refractory gastroparesis.

The GCSI is a widely used, validated assessment of gastroparesis symptoms, but has notable limitations. The total score is calculated by taking the mean of three sub-scores (nausea/vomiting, early satiety, and bloating), but these are weighed unevenly, with bloating items contributing more than the vomiting items, and vomiting items more than early satiety items. Consequently, resolution of abdominal distension in one patient appears to indicate twice the improvement as regaining the ability to finish a meal in another patient. In addition, the GCSI does not assess all the symptoms of gastroparesis, such as abdominal pain. To address this limitation, we also looked at resolution of the predominant gastroparesis symptom, and also found no difference between groups. These findings are consistent with previous studies that have looked at non-GCSI-based symptomatic outcomes after G-POEM and pyloroplasty [22, 23, 27, 29]. The data suggest that both pyloroplasty and G-POEM are equally effective procedures.

One of the defining criteria of gastroparesis is the presence of a delay in gastric emptying. A successful pyloric drainage is expected to improve gastric emptying. In the present study, we found that gastric emptying was significantly improved after both endoscopic and laparoscopic pyloric drainage. However, despite having no difference in preoperative 4 h retention on gastric-emptying scintigraphy, after surgery patients who underwent laparoscopic pyloroplasty had superior emptying, with a trend towards lower 4 h retention and substantially more patients reaching at least 50% improvement in GES and GES normalization. Few studies have directly compared emptying after pyloroplasty and G-POEM. However, this difference in postoperative gastric emptying is evident when the limited single-arm observational literature is assessed in aggregate. Preoperative ranges for 4 h retention on GES are similar in the literature for G-POEM and pyloroplasty. After pyloroplasty, however, studies report the mean 4 h retention ranges from 2.5 to 8.9% [5, 26, 27]. After G-POEM, it is much higher, ranging between 11 and 33% [13–21, 23, 24]. A 4 h retention greater than 10% is considered abnormally delayed. Therefore, the mean 4 h retention after G-POEM in these studies, and in the present study, was all above this threshold, and in some studies, mean 4 h retention after G-POEM exceeds twice the upper limit of normal. By contrast, all the studies demonstrate a mean 4 h retention below the 10% threshold after pyloroplasty. Therefore, laparoscopic pyloroplasty results in a greater improvement in gastric emptying than G-POEM. However, the clinical significance of this finding remains to be demonstrated, as both approaches to pyloric drainage result in significant and comparable symptom improvement. Further, we found no difference in need for gastric stimulator or gastrectomy between groups, suggesting that the patients’ overall outcomes are comparable. However, given these gastric-emptying findings, if a patient had insufficient improvement in symptoms and emptying after G-POEM, a revisional laparoscopic pyloroplasty may prove to be an effective salvage procedure, before progressing to gastrectomy. Further research is necessary to determine the efficacy of revisional surgery after G-POEM.

The present study represents the largest single-center cohort study that directly compares laparoscopic and endoscopic pyloric drainage surgeries with at least 6 months follow-up. Other studies that directly compare pyloroplasty and G-POEM outcomes are limited in both sample size and follow-up, resulting in conflicting conclusions. A study of 63 patients who underwent G-POEM and 48 patients who underwent robotic pyloroplasty found that pyloroplasty resulted in significantly more GCSI score improvement (1.9 vs. 0.9, *p* < 0.001), with lower postoperative GCSI scores (2.0 vs. 2.5, *p* = 0.026) at 1–3 months after surgery [30]. These results contradict the findings of the present study. Not only was pyloroplasty no better than G-POEM, but robotic pyloroplasty was also similar to laparoscopic pyloroplasty. However, there are some notable limitations in their study, which may explain these disparate results. In addition to the limited sample size and short follow-up, patients who underwent robotic pyloroplasty were substantially more likely to have reflux symptoms (79% vs. 13%, *p* < 0.001) managed with proton pump inhibitors (82% vs. 24%, *p* < 0.001). The incompetent lower esophageal sphincter typically associated with gastroesophageal reflux may have a substantial impact on the efficacy of pyloric drainage and explains their findings. Another study directly comparing endoscopic and surgical pyloric drainage looked at 30 patients who underwent pyloroplasty and 30 age, sex, and gastroparesis etiology propensity matched patients who underwent G-POEM. They found that at 90 days after surgery, there was no difference in GCSI score improvement (*p* = 0.85), consistent with our findings. On postoperative GES, however, they found no significant difference between pyloroplasty and G-POEM among the 18 patients who underwent testing (*p* = 0.14), contrary to our conclusion. However, 4 h retention was 2.5% with 100% normalization after pyloroplasty, whereas after G-POEM 4 h retention was 10.7% with only 73% normalization, despite having similar preoperative 4 h retention (36% and 33%, respectively) [5]. Given the small sample size, these GES results likely reflect a type II error, and are consistent with our findings that, despite equivalent symptomatic outcomes, pyloroplasty results in superior improvement in gastric emptying.

The most robust study comparing pyloric drainage surgeries is a meta-analysis by Mohan et al. comprised 332 patients who underwent G-POEM and 375 who underwent pyloroplasty. Consistent with our findings, they found that there was no difference in symptomatic outcomes between surgeries. However, they also found that there was no difference in clinical success based on the 4 h gastric-emptying retention results. Authors concluded that G-POEM outcomes are comparable to pyloroplasty [4]. This conclusion is in contrast to our findings of superior emptying after pyloroplasty. However, the definition they used for a ‘success’ was stated as ‘any improvement in 4 h retention,’ which can be considered as a very generous measure. They reported ‘any improvement’ in 84% of patients who underwent pyloroplasty and in 85.1% of patients who underwent G-POEM (*p* = 0.91). When we applied this definition to our data, we similarly found 85% improvement after pyloroplasty and 83% improvement after G-POEM (*p* = 1.000), despite considerably lower normalization rates of 64% and 46%, respectively. In one study included in this meta-analysis, 33 patients underwent G-POEM and the mean gastric retention at 4 h improved from 45.0 to 29.6% (*p* = 0.04) [17]. Mohan et al. reported this as a 33/33 (100%) success for GES, despite the fact that the mean 4 h retention after intervention remained nearly three times the upper limit of normal, which is 10% retention at 4 h. These data were compared to pyloroplasty studies, such as one which found that 4 h retention decreased from 40.8 to 8.9% after pyloroplasty [26]. However, this was included in the meta-analysis as a 36/40 (90%) success. Clearly, this definition of success does not accurately reflect the actual clinical impact of the pyloric drainage procedure on gastric emptying. Furthermore, despite the conclusion that outcomes were comparable, Mohan et al. actually found that the pooled postoperative 4 h retention after pyloroplasty was 3.9% compared to 20.6% after G-POEM, consistent with our findings that G-POEM has inferior improvement in gastric emptying.

We acknowledge certain limitations of this study including its retrospective nature and non-randomization of surgeries. Early on in our practice, pyloroplasty was the predominant pyloric drainage procedure we used for gastroparesis. Beginning in 2020, our G-POEM practice increased and exceeded pyloroplasty by the end of 2022. Currently, our preference is for G-POEM unless a patient requires anti-reflux surgery, in which case a concurrent pyloroplasty is performed. Despite this evolution in practice, follow-up times after surgery were comparable and when outcomes from surgeries performed before or after the change in practice were compared, there was no difference in the rate of resolution of predominant gastroparesis symptom or GES within the G-POEM or pyloroplasty groups (*p* > 0.05). This finding suggests that evolution of practice did not likely have a strong impact on the results. Our practice also favors pyloric drainage as the initial surgery for gastroparesis. As such, no patients had a prior gastric stimulator in place. We previously reported that gastric stimulator is an effective adjunct after failed pyloric drainage [31]. Further research is necessary to evaluate and compare pyloroplasty and G-POEM as adjuncts to failed gastric stimulator. Another limitation is that, although all patients were offered repeat GES at 1 year after surgery, only 96 patients elected to participate. Some may argue that these patients may represent those with more severe symptoms, which could introduce a selection bias. However, when we compared patients who underwent postoperative GES with those who did not, there was no significant difference in symptomatic outcome. Therefore, the GES results likely represent a true difference between surgeries. Nevertheless, since the finding that pyloroplasty offers superior gastric emptying over G-POEM is supported primarily by single-arm observational studies, external validation with direct comparative studies is necessary to confirm whether pyloroplasty truly outperforms G-POEM in gastric emptying, as indicated by this study and the existing literature.

Endoscopic surgery is preferred by most physicians and patients, as it is viewed as a less invasive approach that limits hospital stay and risk of complications. However, in the present study, we found that these benefits were marginal. Patients who underwent G-POEM were more likely to be discharged from the hospital on post-op day one, but the average length of stay for both procedures was the same, one day. Of note, however, it is our practice to routinely obtain an upper GI contrast study on postoperative day 1 to rule out leak prior to discharge. This practice likely contributed to our comparable lengths of stay. Many centers perform G-POEM as an ambulatory surgery. Given that only 2 (< 1%) patients had a leak after pyloroplasty and no patients had a leak after G-POEM, our findings suggest that same-day discharge can be considered. Nevertheless, previous studies have reported longer lengths of stay than what we have reported. A Cleveland Clinic matched study of 30 pyloroplasty and 30 G-POEM reported lengths of stay of 4.6 days and 1.4 days, respectively (*p* = 0.003). They attributed this increased hospital stay after pyloroplasty to increased complications (5 out of 30 vs. 1 out of 30, *p* = 0.086) and the potential for surgical site infection, even though they had no difference in readmissions (*p* = 0.232) [5]. In the present study, only 2.6% of patients developed a surgical site infection after pyloroplasty and there was no difference in numbers of complications or readmissions. These differences may be due to sample size as a slightly better powered study with 48 patients who underwent pyloroplasty and 63 who underwent G-POEM reported no difference in complications (13% vs. 19%, respectively, *p* = 0.440), consistent with our study. This study did not report the actual length of stay, but methodologically reported discharge 48 h after pyloroplasty and same-day discharge after G-POEM [30]. A meta-analysis comprised mostly single-arm G-POEM or pyloroplasty studies found that the pooled complication rate was 11.0% and 11.4%, respectively (*p* = 0.95) with no difference in length of stay (1.3–6 days after G-POEM and 2–6.2 days after pyloroplasty) [4]. Therefore, overall, the literature is consistent with our findings that length of stay and complications are similar between procedures. Pyloroplasty and G-POEM are equivalently safe procedures for patients with gastroparesis.

## Conclusion

Society guidelines addressing the surgical management of medically refractory gastroparesis have not made clear recommendations, due to the paucity of studies in the literature with adequate sample size and follow-up duration. In the largest single-center cohort study to date, we demonstrated the safety and efficacy of pyloric drainage surgery in 233 patients who underwent laparoscopic pyloroplasty and 81 patients who underwent G-POEM, with follow-up at 16 and 11 months, respectively. Both approaches demonstrated excellent safety profiles, significant symptom improvement, and enhanced gastric emptying. Our findings support consideration of pyloric drainage surgery for well-selected patients with medically refractory gastroparesis. Comparisons between laparoscopic pyloroplasty and G-POEM revealed no significant differences in hospital length of stay, readmission rates, need for additional interventions, or safety. In addition, symptom improvement was equivalent, with the qualitative and quantitative tools, we currently have to measure Quality-of-Life outcomes. These findings suggest that either approachs may be offered based on patient and surgeon preference. Notably, our data and the current literature indicate that laparoscopic pyloroplasty is associated with superior objective improvement in gastric emptying. However, given the similar outcomes in other domains, the clinical relevance of this difference warrants further investigation.
